# Functional Changes of the Ocular Surface Sensory Nerves Due to Contact Lens Use in Young Symptomatic and Asymptomatic Users

**DOI:** 10.1167/iovs.64.14.12

**Published:** 2023-11-08

**Authors:** José Ángel Pastor-Zaplana, Juana Gallar, M. Carmen Acosta

**Affiliations:** 1 Instituto de Neurociencias, Universidad Miguel Hernández-CSIC, Sant Joan d'Alacant, Spain; 2 Departamento de Patología y Cirugía, Universidad Miguel Hernández de Elche, Sant Joan d'Alacant, Spain; 3 Instituto de Investigación Biomédica y Sanitaria de Alicante, Alicante, Spain

**Keywords:** contact lenses (CL), corneal sensitivity, corneal sensory nerves, blinking, tearing

## Abstract

**Purpose:**

The purpose of this study was to analyze the differences in corneal sensory nerve functionality in young asymptomatic (CL-A) and symptomatic (CL-S) contact lens (CL) users.

**Methods:**

CL wearers (23.8 ± 1.0 years, *n* = 31) were classified as CL-S with an Ocular Surface Disease Index (OSDI) ≥ 13 (*n* = 14) or CL-A. Users of eye glasses (EG; 24.5 ± 0.8 years, *n* = 29) with OSDI < 13 participated as controls. The sensations evoked by mechanical, chemical (gas esthesiometer), and cold (4°C saline drops) stimuli were measured using the Visual Analogue Scales (VASs). Moreover, tear volume, tear break up time (TBUT), blinking frequency (BF), and ocular surface temperature (OST; IR thermography) were also measured.

**Results:**

Mechanical and chemical stimuli produced similar scores in the CL-A and EG participants, although the CL-A subjects referred to stronger irritation (p < 0.05). Likewise, the VAS intensity in response to cold stimuli did not differ between CL-A and EG subjects, while the ability to detect cold was significantly worse in CL-S users (p < 0.05). CL-A users had a similar tear volume, a higher BF (p < 0.01) and shorter TBUT (p < 0.001) to EG wearers, and blinking and TBUT were also altered significantly in CL-S users (p < 0.01). Interestingly, the OST was significantly lower in CL-A users (p < 0.05) than in EG wearers, but not in CL-S users.

**Conclusions:**

Using CLs modifies corneal sensitivity, blinking and tearing in young volunteers. Even if they have yet to develop clinical signs of inflammation, they display changes in corneal sensitivity consistent with the sensitization of corneal nociceptors and the inhibition cold thermoreceptors, phenomena that occur under inflammatory conditions. The differences in corneal sensitivity and OST between CL-A and CL-S users could reflect the extent of nerve damage and inflammation at the ocular surface.

The ocular surface (OS) is innervated by sensory nerves that define its sensitivity, evoking sensations like irritation and pain,^1^^,^^2^ as well as initiating protective reflexes like blinking and tearing.^3^^–^^6^ Sensory nerves are classified based on the type of stimuli they respond to: mechanonociceptors that respond to mechanical forces; polymodal nociceptors recruited by mechanical, heat and chemical stimuli; and cold thermoreceptors that respond to a decrease in the OS temperature (OST) and increases in tear film osmolarity.^1^^,^^7^^–^^9^ As such, the activation of nociceptors evokes irritation and pain, whereas the stimulation of cold thermoreceptors mainly evokes sensations of cooling and dryness.^1^ Cold thermoreceptors can also be subclassified into high background-low threshold (HB-LT) and low background-high threshold (LB-HT) cold thermoreceptors,^8^^,^^10^ both depending on their background activity at normal OSTs and the temperature decrease necessary to increase their background activity. Moreover, there is evidence that HB-LT thermoreceptors are responsible for cooling sensations,^1^ whereas the LB-HT thermoreceptors are responsible for the sensation of dryness and irritation, or of pain, evoked by cold stimulation of the OS.

Cold thermoreceptors are involved in regulating tearing and blinking, and indeed, basal tearing rates and blinking frequencies depend on the sustained background activity of cold thermoreceptors.^5^^,^^6^ However, the endogenous spontaneous blink generator located in the brainstem can be modulated by both afferent sensory input from the OS, and the cognitive state and brain cortex activity.^11^ Moreover, reflex tearing and blinking can both be induced by the activation of corneal nociceptors.^3^^,^^4^^,^^12^

Under conditions of inflammation and after damage to ocular tissues, the activity of sensory nerves is altered,^13^^–^^17^ evoking sensations of irritation and pain, and inducing changes in blinking and tearing.^18^^,^^19^ The remarkable increases in tear film osmolarity that have been proposed to occur with tear film break-up would be sufficient to stimulate cold thermoreceptors and activate polymodal nociceptors, thereby contributing to the ocular discomfort provoked by acute and excessive drying of the OS.^9^^,^^20^ The spontaneous and stimulus-evoked activity of corneal sensory nerves is altered under inflammatory conditions, although each type of sensory nerve is altered in a different way. For example, corneal nociceptors become sensitized and enhance their activity whereas cold thermoreceptors are inhibited by inflammation.^13^^,^^14^

Contact lens (CL) wearing and cleaning solutions produce mechanical forces, temperature changes, and chemical stimulation of the OS, either directly due to exogenous irritation or indirectly through the release of endogenous agents due to cell damage, hypoxia, or changes in pH or osmolarity.^21^ These stimuli will lead not only to sensory nerve stimulation but also to the damage of nerve terminals and local inflammation.^18^ In turn, these events further activate and sensitize sensory nerves, evoking the discomfort and pain reported by some CL users.^21^

The effects of using CLs of different types and composition have been studied on different facets of the OS, focusing on the corneal epithelium, corneal nerve morphology and density, corneal sensitivity, corneal surface temperature, tear film, blinking, etc.^21^^–^^50^ Yet how these effects of CL use influence the activity of the different functional types of corneal sensory nerves, or their sensitive and protective roles, has not yet been addressed. Accordingly, the relationship between nerve activity and the sensations and/or symptom’s evoked by CL use remains unclear. Hence, the aim of this study was to analyze the changes in the function of corneal sensory nerves in young asymptomatic CL users (CL-A), and to compare these to those in age-matched asymptomatic eyeglass (EG) wearers and symptomatic CL users (CL-S). Exploring the sensitivity to selective mechanical, chemical, and cold stimulation of the cornea revealed some changes in corneal sensitivity that could be attributed to alterations induced by inflammation. Measuring the OST provided evidence of inflammation, even in the absence of clinical signs. The differences in sensitivity to selective stimulation of CL-A and CL-S users suggested different corneal conditions that might explain the appearance of symptoms.

## Methods

### Subjects

Young volunteers of both sexes participated in this study (*n* = 31), 7 men and 24 women between 18 and 40 years of age (mean 23.81 ± 0.95 years) who use soft, monthly CLs (hydrogel or silicon hydrogel, 36–67% water) for at least 8 hours daily (CL group). In addition, 29 asymptomatic EG wearers of a similar age and with no OS symptoms (Ocular Surface Disease Index [OSDI] D < 13) participated as a control group. All the participants were students and staff of the Universidad Miguel Hernández (Spain) and the study was carried out in accordance with the tenets of the Helsinki Declaration. All the volunteers provided their signed informed consent prior to participating and the study protocol was approved by the Ethics Committee at our university (Comité de Ética e Integridad en la Investigación de la Universidad Miguel Hernández de Elche). None of the participants had a history of corneal or ocular disease.

The OSDI was recorded for all the participants and based on their total OSDI value (OSDI D), CL users were classified as asymptomatic (OSDI < 13) or symptomatic (≥ 13).^51^^,^^52^ All the EG subjects selected for the control group had an OSDI < 13. The OSDI measurements were all made by the same researcher in a room at a controlled temperature (22.36 ± 0.98°C) and humidity (39.38 ± 5.91%).

Of the 31 CL users, 14 (45.2%) were classified as CL-S and 17 (54.8%) as CL-A based on their OSDI D scores (see the Table for the characteristics of these participants). Both CL-A and CL-S users had been using contact lenses for a similar time (see the Table) and, whereas the CL users required a stronger refractive correction, the refractive index was not considered an exclusion factor for the study (see the Table).

**Table.** Characteristics of the Study Participants

	Eye Glasses	CL Asymptomatic	CL Symptomatic
**Age** **,** **y**	24.5 ± 0.8	24.3 ± 1.4	23.2 ± 1.3
**Gender**	9 men; 20 women	2 men; 15 women	5 men; 9 women
**Refractive error (diopters)**	−2.33 ± 0.17	−5.40 ± 0.72**	−3.61 ± 0.31**
	[−0.25 to −5.50]	[−1.75 to −15.5]	[−1 to −5.5]
**Time of CL use** **,** **y**	10.1 ± 1.4	9.2 ± 1.4	7.8 ± 1.1
**Medications**	Oral contraceptives (*n* = 6)	Oral contraceptives (*n* = 2)	Oral contraceptives (*n* = 2)
***n***	29	17	14

The data are the means ± SEM. For the refractive error, both the mean ± SEM and the range (in brackets) are shown: **P < 0.001, Mann-Whitney *U* test, relative to eye glass wearers. No differences were found between the CL-A and CL-S subjects either for refractive error (*P* = 0.169) or other parameters.

After completing the OSDI questionnaire, different measurements were acquired in the following order: blinking frequency at rest, attentional blinking frequency, OST (2 minutes after CL removal), corneal sensitivity, tear volume, and tear break-up time (TBUT). In most cases, both eyes were explored, except when assessing corneal sensitivity, which was only assessed after stimulating the right eye.

### Blinking Frequency

Blinking frequency was assessed over 1 minute from video recordings of the volunteers’ face. Both the spontaneous blinking frequency under basal conditions (blinking frequency at rest) and the attentional blinking frequency while performing a D2 attentional test for Spanish-speaking people^53^ were measured. In the D2 test, the subject is presented with 14 printed lines in which the letter “d” appears repeatedly, interspersed with the letter “p.” Some of these letters are accompanied by one or two short lines, located in different positions around the letter. The subject must mark each letter “d” that has two dashes distributed in a defined position, selecting the “relevant” stimuli and inhibiting the “irrelevant” ones. The time allotted to complete each line is 20 seconds and the blinking frequency was measured during the first minute of this attentional task.

### Ocular Surface Thermography

The OST was measured in both of the volunteer's eyes in videos taken with an infrared video camera (InfRec R300SR; Nippon Avionics). Subjects were asked to close their eyes for 10 seconds and they were then filmed for 1 minute (at 60 frames/s) after opening their eyes. They could blink freely during the recording and the images were analyzed offline using dedicated software (InfReC Analyzer NS9500 Standard, version 2.7) in order to define the evolution of temperature over time. OST was analyzed in 3 OS regions 4 seconds after eye opening: the central cornea, a circular area of 30 pixels in the center of the cornea; the nasal conjunctiva, a 16 pixel oval area; and the temporal conjunctiva, an 8 pixel oval area. Conjunctival temperature was also measured in order to detect possible increases in temperature due to conjunctival hyperemia. In CL users, the OST was recorded 2 minutes after the CL removal. The average data from both eyes of each subject were pooled for each group (CL-S, CL-A, and EG).

### Corneal Sensitivity

A CRCERT-Belmonte esthesiometer^54^^,^^55^ was used to measure mechanical and chemical corneal sensitivity. Over a controlled period, this instrument delivers a jet of gas to the eye that contains a mixture of air and CO_2_ in specific proportions, and at a flow rate between 0 and 200 mL/min. A modified optical range finder in the probe was used to maintain the distance of 4 mm between the probe and the eye, and the gas was warmed inside the probe to reach the surface of the cornea at a temperature of 34°C.^1^^,^^54^^–^^56^

For each subject, corneal sensitivity was measured in only one eye, delivering 3-second pulses of gas with a 2-minute interval between each of them. For mechanical stimulation, fixed temperature pulses were delivered to the central cornea at different flow rates (40, 80, 120, 160, and 200 mL/min). For chemical stimulation, the proportion of CO_2_ in air was varied (40, 60, and 80%) and the pulses were delivered at a flow rate of 40 mL/min. The sensitivity to intense cold was also explored by instilling the eye with a 60 µL drop of saline at 4°C.

After each stimulus (gas pulse or cold saline drop), the intensity of the sensation evoked in the subjects was scored, as was the irritation component, using 2 separate 10 cm Visual Analog Scales (VASs), whereby 0 was no sensation and 10 the maximum expected sensation. The cold sensation after stimulation was also evaluated with a separate VAS.

A minimum sample size was calculated to achieve a power of 0.90 and *P* = 0.05, based on the differences of the means and SDs obtained in our previous studies,^56^ and this was 10 subjects.

### Tearing and TBUT

Tearing and TBUT were measured in both eyes of each subject and the data were pooled. The tear volume was measured using phenol red threads (Zone-Quick; Menicon, Tokyo, Japan) that were placed in the temporal cantus of the lower lid for 15 seconds. The length of the red thread wetted (in mm) was measured.

To determine the TBUT, a commercial fluorescein strip (Optitech Eyecare, Prayagraj, India) moistened with sterile saline solution was applied to the eye surface. The subject was then asked not to blink while the tear film was observed under the broad beam of a slit-lamp using cobalt-blue light. The TBUT was recorded as the time in seconds that elapsed between the blink and the appearance of the first dry spot in the tear film.

### Statistical Analysis

Statistical analysis was carried out using SigmaPlot version 11.0 (Systat Software, Inc.). This software initially analyzes the normality of the data and then applies the necessary parametric or nonparametric test. The data are expressed as the mean ± SEM or as the median and interquartile range (IQR), and they were compared using a parametric or equivalent nonparametric test as necessary, and as indicated in the text, tables, and figures. The significance level was set as *P* < 0.05 in all the statistical analyses.

## Results

### Corneal Sensitivity

#### Sensitivity to Mechanical Stimulation

**Figure 1.**
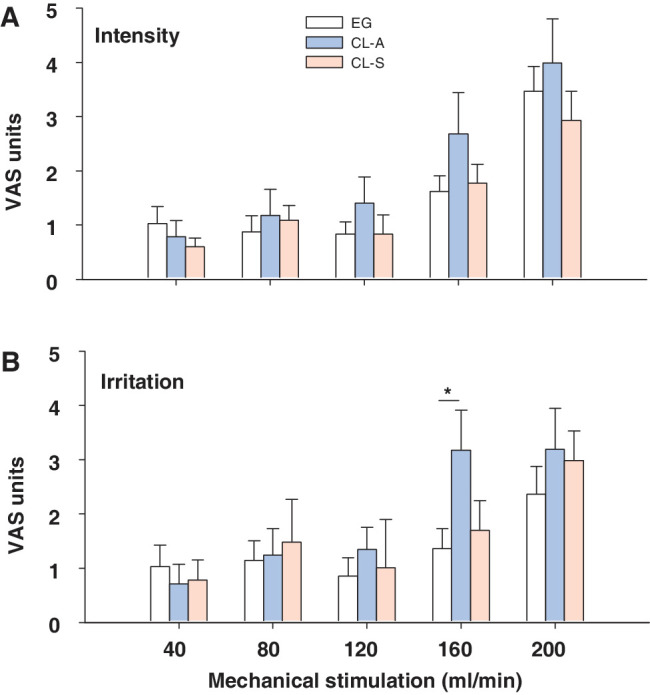
Intensity (**A**) and irritation (**B**) VAS values reported following mechanical stimulation of the central cornea. Jets of warm air at different flow rates (mL/min) were applied to the cornea of EG wearers and CL users, the latter classified as asymptomatic (CL-A) or symptomatic (CL-S) depending on their OSDI scores (see Methods). The data are the mean ± SEM: *P < 0.05, Mann-Whitney *U* test.

There were no significant differences between the EG and CL-A or CL-S subjects in terms of the VAS intensity values obtained in response to mechanical stimulation, although above a flow rate of 80 mL/min, the intensity of the CL-A VAS values were always higher than those of the other 2 groups (Fig. 1A). In terms of irritation, the VAS values evoked in the CL-A subjects achieved a maximum at a rate of 160 mL/min and this did not increase with the maximal stimulus intensity of 200 mL/min, in contrast to the progression in the EG or CL-S subjects (see Fig. 1A). The VAS values for irritation in the CL-S subjects were generally slightly higher than those recorded by the EG subjects, but not as high as those evoked in the CL-A subjects (Fig. 1B; see Supplementary Table S1 for the detailed statistical analysis).

#### Sensitivity to Chemical Stimulation

**Figure 2.**
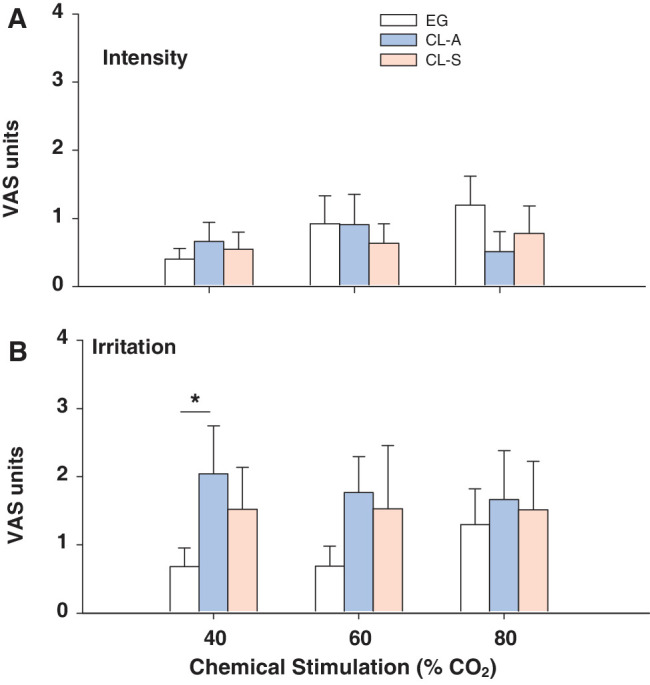
Intensity (**A**) and irritation (**B**) VAS values reported following chemical stimulation of the central cornea of EG wearers and symptomatic (CL-S) or asymptomatic (CL-A) CL users. The data are the mean ± SEM: *P < 0.05, Mann-Whitney *U* test.

As for mechanical sensitivity, no significant changes were observed in the VAS intensity values reported following chemical stimulation in either type of CL user relative to the EG wearers (Fig. 2A). However, 40% CO_2_ did evoke the maximal VAS irritation response in CL-A and CL-S users but not in EG wearers (Fig. 2B; see Supplementary Table S2 for the detailed statistical analysis).

#### Sensitivity to Cold Stimulation

**Figure 3.**
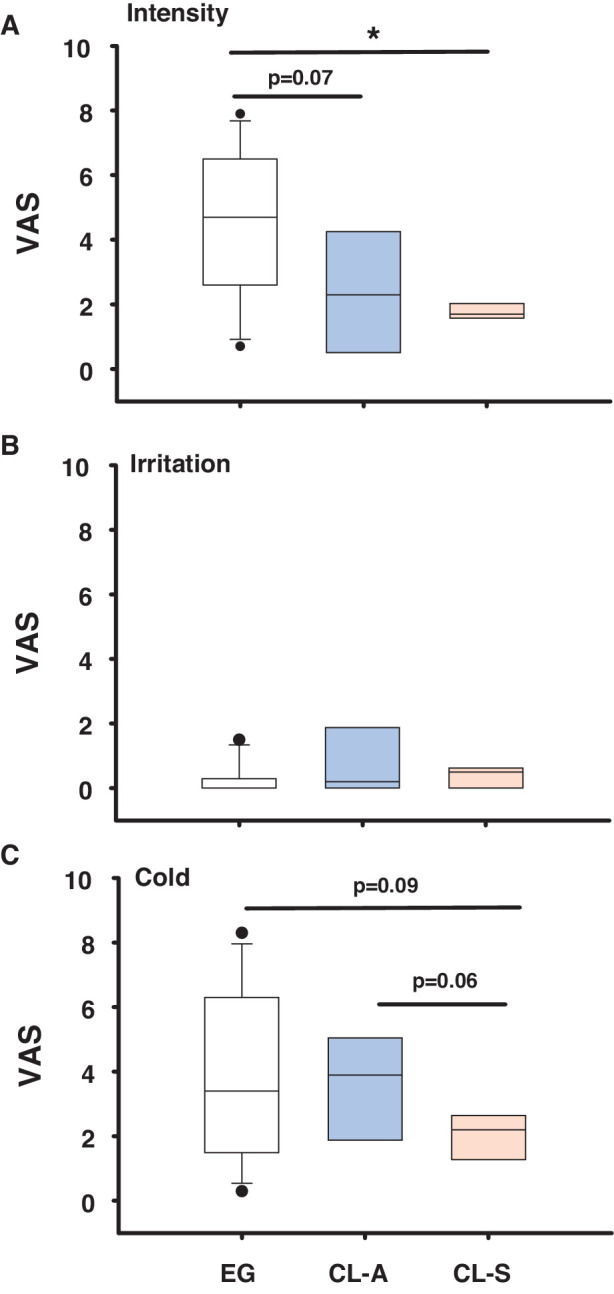
Box plots showing the VAS values reported for the intensity (**A**), irritation (**B**), and cold component (**C**) of the sensation evoked by instilling a cold saline (4°C) drop onto the eye in eye glass (EG) wearers and CL symptomatic (CL-S) or asymptomatic (CL-A) CL users. The boxes cover the 25th to 75th percentiles, the central line is the median, and the bars reflect the 10th and 90th percentiles: *P < 0.05, Mann-Whitney *U* test.

A drop of cold saline (4°C) was delivered to the subjects’ eye to induce intense cooling of the OS and this cold stimulation evoked sensations rated with lower VAS intensity values by CL users relative to EG wearers, especially in the CL-S group (Fig. 3A). Similarly, the VAS cooling values reported after cold stimulation were also lower in CL-S than in CL-A subjects (Fig. 3C). Notably, the VAS values of irritation evoked by cold stimulation were very small in all the subjects explored (Fig. 3B; see Supplementary Table S3 for the detailed statistical analysis).

### Spontaneous and Attentional Blinking Frequency

**Figure 4.**
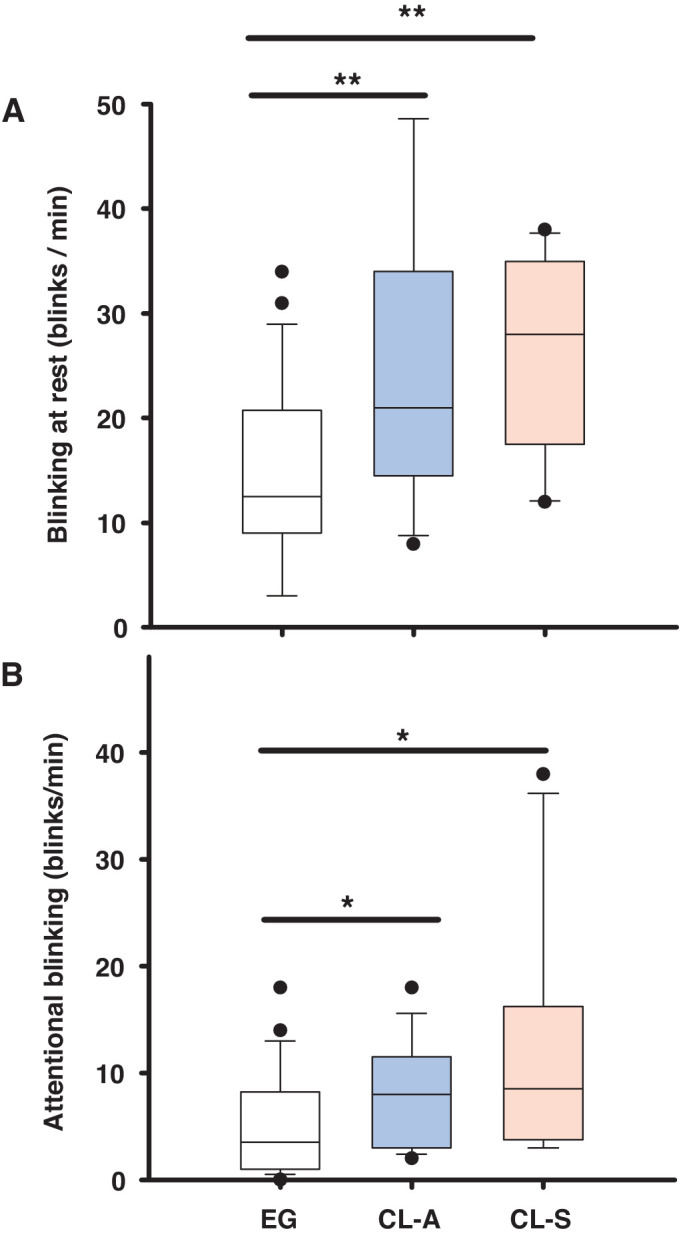
Box plots showing the blinking frequency at rest (**A**), and during the performance of an attentional task (attentional blinking) (**B**), in the EG wearers and CL users: symptomatic (CL-S) and asymptomatic (CL-A). The boxes cover the 25th to 75th percentiles, the central line is the median, and the bars reflect the 10th and 90th percentiles: **P < 0.01, *P < 0.05, *t*-test or Mann-Whitney *U* test.

The blinking frequency at rest (spontaneous blinking frequency) was significantly higher in both CL-A and CL-S users than in EG wearers (Fig. 4A). During the performance of an attentional task, blinking frequency was significantly lower than at rest in all groups, yet it was higher in CL-A and CL-S users than in EG wearers (Fig. 4B; see Supplementary Table S4 for the detailed statistical analysis).

### Tearing and TBUT

**Figure 5.**
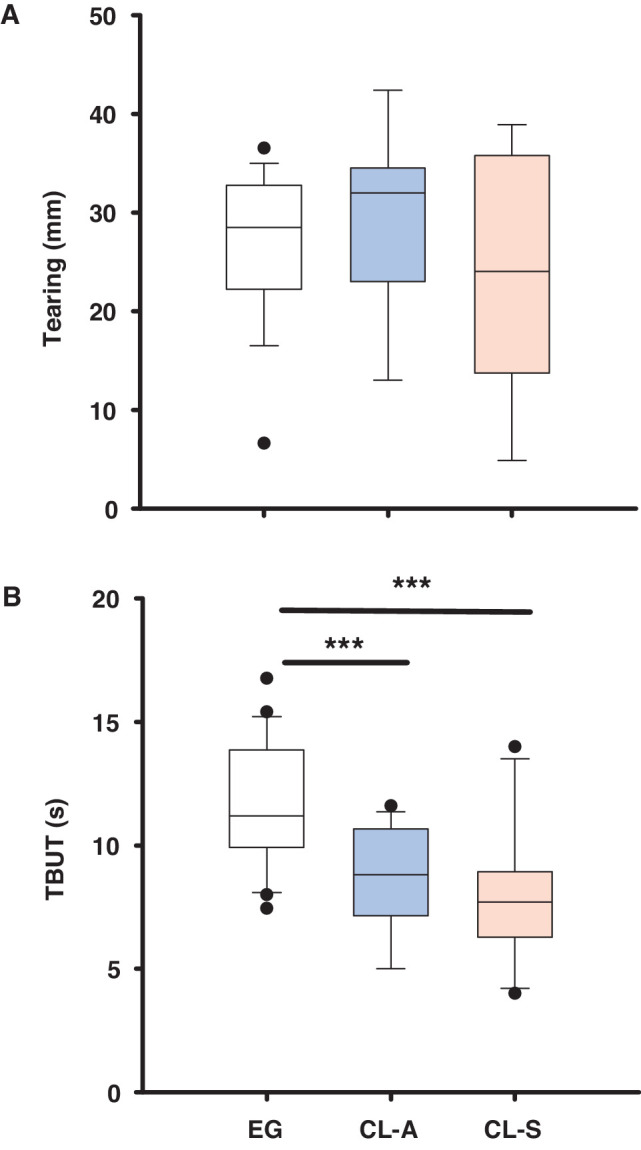
Box plots showing the tear volume (**A**) and TBUT (**B**) in EG wearers and CL users. The boxes cover the 25th to 75th percentiles, the central line is the median, and the bars reflect the 10th and 90th percentiles: **P < 0.001, *t*-test.

No significant differences were found in the tear volume of EG wearers and CL users (Fig. 5A and see Supplementary Table S4 for the detailed statistical analysis). By contrast, the TBUT was significantly shorter in CL users than in EG wearers (Fig. 5B and see Supplementary Table S4 for the detailed statistical analysis).

### Ocular Surface Temperature 

**Figure 6.**
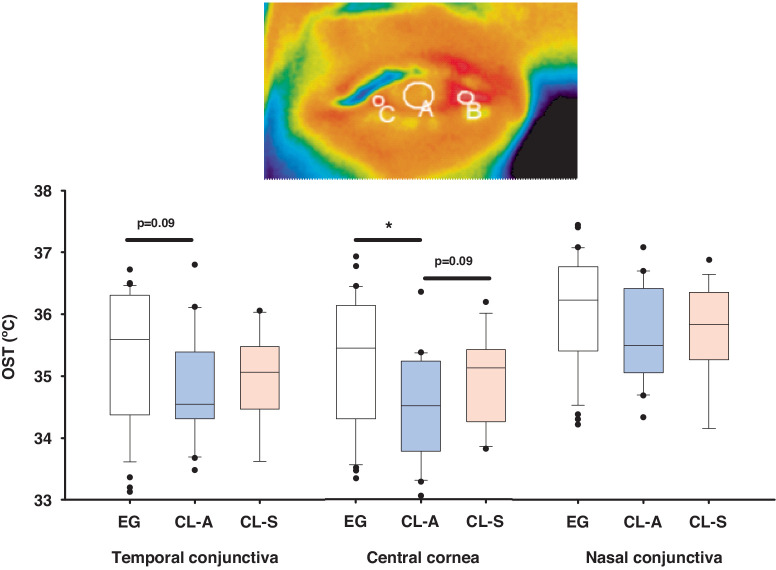
Box plots showing the ocular surface temperature (OST) in the central cornea (**A**), and the nasal (**B**), and temporal (**C**) conjunctiva, measured from infrared thermographic images of EG wearers, and asymptomatic (CL-A) and symptomatic (CL-S) CL users (2 minutes after CL removal). The data are the mean ± SEM and the boxes cover the 25th to 75th percentiles, the central line is the median, and the bars reflect the 10th and 90th percentiles: *P < 0.05, *t*-test.

The OST was measured in the central cornea, and in the temporal and nasal conjunctiva (Fig. 6), and the OST values from the central cornea were significantly lower in CL-A users than EG wearers, and they were slightly lower in the temporal and nasal conjunctiva of CL-A users (*P* = 0.09 and *P* = 0.162, respectively; see Fig. 6). In general, CL-S users also had lower OST values than EG wearers, but higher than CL-A users (Fig. 6 and see Supplementary Table S5 for the detailed statistical analysis).

## Discussion

We show here how the use of CL alters the function of OS sensory nerves and modifies corneal sensitivity, thereby altering blinking and tearing. Moreover, the differences evident between subjects with and without symptoms of ocular discomfort could be attributed to a different degree of OS inflammation or damage in each situation, both of which are known to affect the activity of OS sensory nerves.^13^^,^^14^^,^^16^^,^^17^

Although it has often been reported that CL users do not experience significant changes in corneal nerve morphology or density,^33^^,^^34^^,^^39^^,^^47^^,^^57^ corneal sensitivity is significantly diminished in these individuals irrespective of the type or composition of the CLs they use.^21^^–^^27^^,^^30^^,^^32^^,^^33^^,^^38^^,^^41^^,^^47^ This loss of corneal sensitivity has been proposed to be the result of hypoxia and/or mechanical trauma induced by CL use,^25^^–^^27^^,^^32^^,^^33^^,^^38^^,^^58^^–^^60^ although adaptation to mechanical stimulation due to CL use has been also proposed.^61^ The data here indicate that young CL and EG users report similarly intensity values for the sensations evoked by both mechanical and chemical stimulation with a gas esthesiometer, irrespective of whether they have ocular symptoms or not. However, the reported VAS values for irritation were higher in CL users than in EG, particularly in CL-A users. By contrast, the intensity values reported by CL wearers after cold stimulation were lower, and in this case particularly in CL-S subjects. These data on corneal sensitivity in humans fit perfectly with the changes observed in the activity of corneal sensory nerves recorded in injured or inflamed guinea pig corneas. Sensitization of corneal nociceptors, which would lead to an increase in the irritation component of the evoked sensations, occurs in damaged, inflamed, and tear-deficient corneas.^13^^–^^17^ Conversely, the inhibition of cold thermoreceptor activity, which would explain the decrease in sensitivity to cold stimulation, is also observed in inflamed corneas.^13^^,^^14^^,^^17^

CL use may induce both ocular surface inflammation and corneal nerve lesion,^21^ although the mechanisms behind these effects remain unclear.^18^ The results presented here support the idea that the altered corneal sensitivity evident in CL users depends on the extent of damage and/or inflammation induced by CL use, and, hence, on the changes induced in nerve activity. During repetitive nerve stimulation and after lesion of the OS, both mechano- and polymodal nociceptors are sensitized,^16^^,^^17^ and their enhanced response to natural stimulation explains the increased irritation and sensations of discomfort experienced under these conditions. When inflammation of the OS is induced, desensitization of cold thermoreceptors is also produced.^13^^,^^14^^,^^17^ The weaker response to cooling of cold thermoreceptor nerves in inflamed corneas explains the dampened sensitivity to cold under inflammatory conditions. Accordingly, the data presented suggest that mild damage to corneal nerves and very mild local inflammation is induced in CL-A subjects. Under these conditions, sensitization of corneal nociceptors is expected to be the main change in corneal nerve activity.

Apart from a mild nerve affectation, more prominent inflammation would also be induced by CL use in CL-S subjects, albeit still subclinical in most cases, as suggested by their higher mean OST than in CL-A subjects. Under these conditions, both the sensitization of nociceptors and inhibition of cold thermoreceptors would be expected, which fully explains the stronger irritation component evoked by mechanical and chemical stimulation, and the lower sensitivity to cold stimulation observed in CL-S subjects. This hypothesis is also sustained by the accumulation of inflammatory mediators (such as cytokines, NGF, SP, etc.) and the density of immune cells (such as dendritic and Langerhans's cells) described in CL users,^21^^,^^39^^,^^62^^–^^66^ especially CL-S users.

The activity of corneal sensory nerves is implicated in the control of blinking and tearing, both of which serve as protective mechanisms. Polymodal nociceptor activity is responsible for reflex blinking^3^ and tearing,^4^ whereas the activity of cold thermoreceptors is responsible for basal tearing^5^ and blinking.^6^ We found CL users had a higher blinking frequency, consistent with the sensitization and enhanced activity of polymodal nociceptors. This increased blinking frequency was observed both at rest and during visual attention, which suggests a regulation of blinking to protect the eye from an adverse environment or desiccation. Hence, it appears that the sensory input provided to the brainstem by trigeminal neurons innervating the cornea prevails over the descending modulation exerted by the brain cortex to reduce blinking while performing a task requiring visual attention.^3^^,^^12^

In terms of the tearing rate, there was no significant change in tear volume in CL users, as reported previously,^35^ although there was a large variability in tear volume within these subjects, especially those who were symptomatic. No significant changes in tearing rate have been observed in animals with mild corneal inflammation.^14^ We did observe a significant reduction in TBUT in CL users, in line with the previously reported disruption of the tear film due to a thinner lipid layer in CL users.^31^^,^^67^ These changes to the lipid layer augment evaporation and increase tear osmolarity,^28^ with the latter increasing the activity of both cold thermoreceptors and polymodal nociceptors depending on the osmolarity maintained: cold thermoreceptors are activated by small increases in osmolarity, while large increases of tear osmolarity activate polymodal nociceptors and silence cold thermoreceptors.^9^ These responses seem to be produced in the case of the CL users, especially in those who are symptomatic.

OST was proposed as an objective measure of tear film stability, as both these parameters are strongly correlated.^46^^,^^68^ OST values are also thought to reflect OS inflammation given that OST values correlate strongly with the degree of hyperemia of the bulbar conjunctiva.^69^ We found significantly lower OST values in CL users, confirming previous findings demonstrating that such OST changes are induced by CL use and not dependent on CL composition.^36^^,^^37^^,^^46^ The increased evaporation rate when using a CL is thought to underlie the lower OST in CL users,^37^ which is independent of the CL water composition.^28^ The OST was higher in CL-S than in CL-A users, most probably reflecting the inflammation in these symptomatic subjects.^69^ Moreover, although we did not measure the inflammation directly, it is already known that CL wear is intrinsically inflammatory,^70^ even with soft lenses, inducing the expression of pre-inflammation markers.^71^ Indeed, even soft CL use provokes a chronic, low grade, subclinical inflammatory status of the anterior eye called “para-inflammation.” This low-grade inflammatory response to tissue stress can be considered to exist between the basal homeostatic state and symptomatic inflammation.^70^

**Figure 7.**
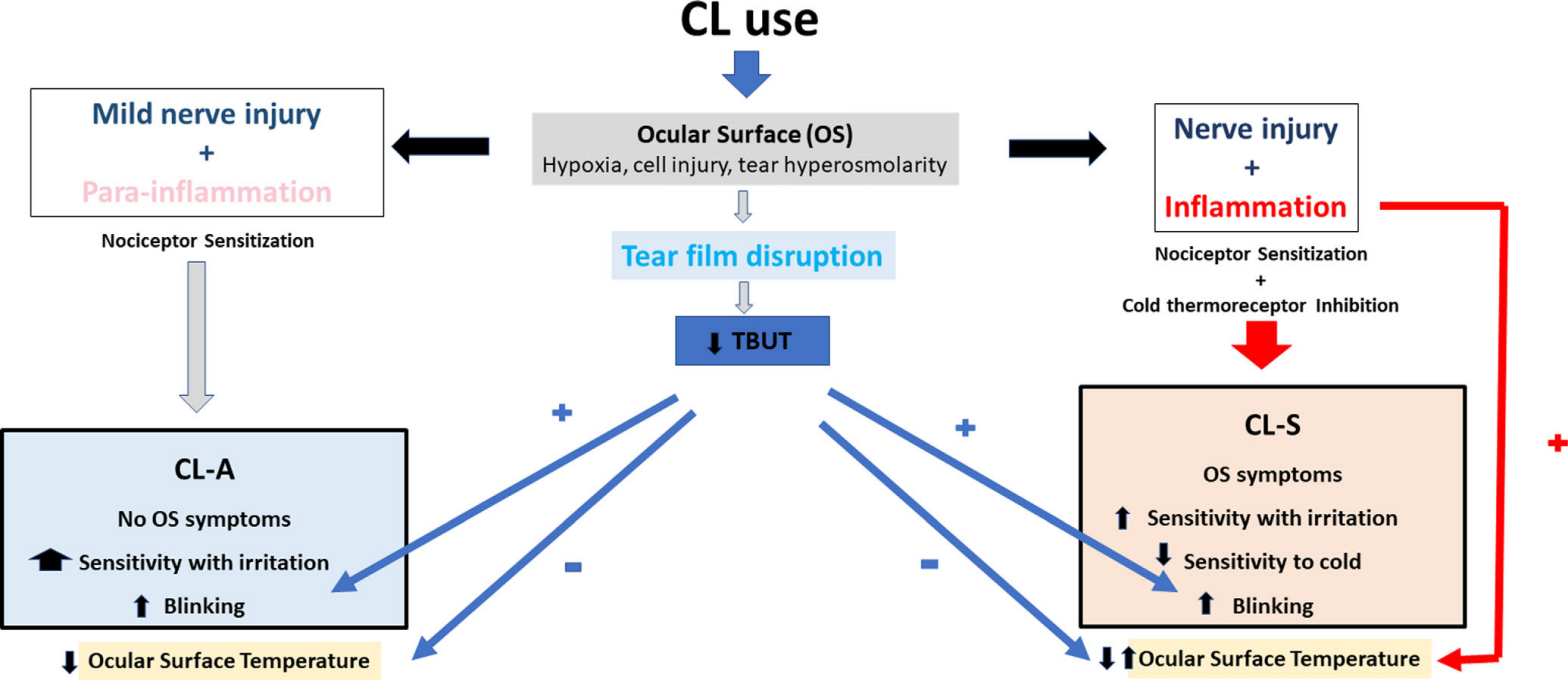
Graphical abstract summarizing the effects of CL use on corneal sensitivity, blinking frequency, TBUT, and the development of ocular surface symptoms.

In conclusion, CL use may induce different degrees of OS damage and inflammation that will affect the activity of corneal sensory nerves. These conditions induce changes in corneal sensitivity and, consequently, they alter the processes driven by sensory input like blinking and tearing. The corneal sensitivity of CL-A subjects suggest that CL use only mildly sensitizes nociceptors, which enhances the sensation of irritation and blinking frequency but does not induce OS symptoms. By contrast, CL use can produce more intense tissue damage and inflammation in symptomatic subjects that, while remaining subclinical, is associated with a higher OST, and the ensuing sensitization of nociceptors and inhibition of cold thermoreceptors. Accordingly, both the irritation component of the sensations experienced and the blinking frequency increase in CL-S subjects, dampening their sensitivity to cold stimulation and producing OS symptoms (Fig. 7).

## Supplementary Material

Supplement 1
