# Supplementary material for: Functional Changes of the Ocular Surface Sensory Nerves Due to Contact Lens Use in Young Symptomatic and Asymptomatic Users
Source: Invest Ophthalmol Vis Sci. 2023 Nov 8;64(14):12. doi: 10.1167/iovs.64.14.12 (PMC10637199; doi:10.1167/iovs.64.14.12)
Supplement: Supplement 1 [file iovs-64-14-12_s001.pdf]

TABLE S1. Intensity and irritation VAS values scored to mechanical stimuli applied to the central cornea. Jets of warm air at different flows (ml/min) were applied to the cornea of eyeglasses users (EG) and asymptomatic (CL-A) or symptomatic (CL-S) contact lens wearers.

	**Intensity (VAS value)**				**Irritation (VAS value)**			
**Air flow (ml/min)**	**EG**	**CL-A**	**CL-S**	***p value*** ***EG vs CL-A*** ***EG vs CL-S*** ***CL-A vs CL-S***	**EG**	**CL-A**	**CL-S**	***p value*** ***EG vs CL-A*** ***EG vs CL-S*** ***CL-A vs CL-S***
**40**	1.02±0.32	0.78±0.30	0.60±0.16	*0.803* *0.804* *0.808*	1.03±0.39	0.71±0.36	0.78±0.37	*0.384* *0.758* *0.743*
**80**	0.87±0.30	1.18±0.48	1.09±0.28	*0.609* *0.186* *0.552*	1.14±0.36	1.24±0.49	1.48±0.78	*0.971* *0.865* *0.793*
**120**	0.84±0.22	1.41±0.48	0.84±0.35	*0.341* *0.813* *0.631*	0.85±0.33	1.35±0.41	1.01±0.89	*0.253* *0.606* *0.200*
**160**	1.62±0.29	2.68±0.77	1.77±0.35	*0.439* *0.759* *0.685*	1.36±0.36	3.18±0.73	1.70±0.54	***0.031*** *0.678* *0.140*
**200**	3.46±0.46	3.99±0.81	2.93±0.53	*0.720* *0.493* *0.320*	2.36±0.52	3.19±0.75	2.98±0.54	*0.310* *0.297* *0.381*

Data are mean ± SEM. Comparisons were done using t-test or Mann-Whitney test, depending on the normality of the data. EG, n=22; CL-A, n=13; CL-S, n=10.

TABLE S2. Intensity and irritation VAS values scored to chemical stimuli applied to the central cornea. Jets of warm air containing different percentages of CO_2 were applied to the cornea of eyeglasses users (EG) and asymptomatic (CL-A) or symptomatic (CL-S) contact lens wearers.

	**Intensity (VAS value)**				**Irritation (VAS value)**			
**% of CO_2**	**EG**	**CL-A**	**CL-S**	***p value*** ***EG vs CL-A*** ***EG vs CL-S*** ***CL-A vs CL-S***	**EG**	**CL-A**	**CL-S**	***p value*** ***EG vs CL-A*** ***EG vs CL-S*** ***CL-A vs CL-S***
**40**	0.40±0.16	0.66±0.28	0.55±0.25	*0.211* *0.598* *0.742*	0.68±0.27	2.04±0.71	1.52±0.62	***0.043*** *0.277* *0.544*
**60**	0.92±0.41	0.91±0.44	0.64±0.28	*0.978* *0.932* *0.961*	0.69±0.26	1.77±0.53	1.53±0.92	*0.121* *0.263* *0.491*
**80**	1.19±0.43	0.51±0.30	0.78±0.40	*0.205* *0.733* *0.376*	1.29±0.53	1.67±0.72	1.51±0.72	*0.553* *0.599* *0.879*

Data are mean ± SEM. Comparisons were done using t-test or Mann-Whitney test, depending on the normality of the data. EG, n=22; CL-A, n=13; CL-S, n=10.

**TABLE S3. Intensity, irritation and cold sensation VAS values scored to cold stimuli applied to the central cornea.** A drop of saline at 4ºC was applied to the cornea of eyeglasses users (EG) and asymptomatic (CL-A) or symptomatic (CL-S) contact lens wearers.				
	**EG**	**CL-A**	**CL-S**	***p value***<br>***EG vs CL-A***<br>***EG vs CL-S***<br>***CL-A vs CL-S***
**Intensity**<br>(VAS units)	4.50±0.70	2.53±0.69	1.77±0.1	*0.067*<br>***0.012***<br>*0.228*
**Irritation**<br>(VAS units)	0.25±0.14	0.75±0.34	0.38±0.13	*0.312*<br>*0.276*<br>*0.385*
**Cold sensation**<br>(VAS units)	3.86±0.73	3.98±0.77	1.95±0.44	*0.919*<br>*0.09*<br>*0.058*
Data are mean ± SEM. Comparisons were done using t-test or Mann-Whitney test, depending on the normality of the data. EG, n=14; CL-A, n=8; CL-S, n=6.				

**TABLE S4. Blinking frequency, tear volume and TBUT of eyeglasses users (EG) and asymptomatic (CL-A) or symptomatic (CL-S) contact lens wearers.**				
	**EG**	**CL-A**	**CL-S**	***p value***<br>***EG vs CL-A***<br>***EG vs CL-S***<br>***CL-A vs CL-S***
**Blinking frequency at rest (blinks/min)**	14.3±1.9	24.5±3.7	25.9±2.9	***0.009***<br>***0.002***<br>*0.772*
**Attentional blinking frequency (blinks/min)**	4.8±1.0	7.7±1.3	11.9±3.4	***0.042***<br>***0.016***<br>*0.347*
**Tear volume (mm)**	27.2±1.6	29.2±2.6	24.2±3.8	*0.479*<br>*0.663*<br>*0.269*
**TBUT (s)**	11.7±0.5	8.7±0.6	7.9±0.8	***<0.001***<br>***<0.001***<br>*0.416*
Data are mean ± SEM. Comparisons were done using t-test or Mann-Whitney test, depending on the normality of the data. EG, n=24; CL-A, n=13; CL-S, n=10.				

TABLE S5. Ocular surface temperature from IR images obtained from eyeglasses users (EG) and asymptomatic (CL-A) or symptomatic (CL-S) contact lens wearers. Temperature was measured in the central cornea and the temporal and nasal conjunctiva.

OCULAR SURFACE TEMPERATURE (ºC)				
	**EG**	**CL-A**	**CL-S**	***p value***<br>***EG vs CL-A***<br>***EG vs CL-S***<br>***CL-A vs CL-S***
**Temporal conjunctiva**	35.30±0.18	34.81±0.19	34.98±0.19	*0.086*<br>*0.159*<br>*0.541*
**Central cornea**	35.21±0.18	34.53±0.18	34.99±0.18	***0.015***<br>*0.346*<br>*0.086*
**Nasal conjunctiva**	36.00±0.16	35.64±0.17	35.70±0.21	*0.150*<br>*0.162*<br>*0.829*

Data are mean ± SEM. Comparisons were done using t-test or Mann-Whitney test, depending on the normality of the data. EG, n=36; CL-A, n=20; CL-S, n=16.
